# Creatine Kinase MB Isoenzyme Is a Complementary Biomarker in Amyotrophic Lateral Sclerosis

**DOI:** 10.3390/ijms241411682

**Published:** 2023-07-20

**Authors:** Natsinee Kittipeerapat, Rachel Fabian, Sarah Bernsen, Patrick Weydt, Sergio Castro-Gomez

**Affiliations:** 1 Department of Neurodegenerative Diseases/Neurology, University Hospital Bonn, 53127 Bonn, Germany; 2 German Center for Neurodegenerative Diseases (DZNE), 53127 Bonn, Germany; 3 Institute of Physiology II, University Hospital Bonn, 53115 Bonn, Germany

**Keywords:** amyotrophic lateral sclerosis (ALS), creatinine kinase (CK), CK-MB isoenzyme, biomarker, prognostic value

## Abstract

Amyotrophic lateral sclerosis (ALS) is an invariably fatal neurodegenerative disease with limited therapeutic options. There is an urgent need for novel biomarkers to be used as surrogates for new therapeutic trials and disease monitoring. In this study, we sought to systematically study creatine kinase isoenzyme MB (CK-MB) in a real-world cohort of ALS patients, assess the diagnostic performance, and evaluate its association with other laboratory and clinical parameters. We reviewed data from 194 consecutive patients that included 130 ALS patients and 64 disease control patients (primary lateral sclerosis [PLS], benign fasciculations syndrome [BFS], Huntington’s disease [HD] and Alzheimer’s disease [AD]). CK-MB was elevated in the sera of more than half of all patients with ALS. In patients with spinal-onset ALS, CK-MB levels were significantly higher than in patients with other neurodegenerative diseases. Patients with slower rates of functional decline had a significantly higher baseline CK-MB. Furthermore, CK-MB elevations correlated with cardiac troponin T (cTnT) and with revised ALS Functional Rating Scale (ALSFRS-R) bulbar subcategory. We posit that measuring CK-MB in ALS patients in a complimentary fashion could potentially aid in the diagnostic workup of ALS and help discriminate the disease from some ALS mimics and other neurodegenerative diseases. CK-MB levels also may provide valuable prognostic information regarding disease aggressiveness as well as correlations with specific phenotypic presentations.

## 1. Introduction 

Amyotrophic lateral sclerosis (ALS) is a devastating disease characterized by the progressive degeneration of upper and lower motor neurons [1]. Clinically, this results in a combination of muscle weakness and muscle wasting as a manifestation of lower motor neuron involvement and spasticity as a manifestation of upper motor neuron involvement. The course is invariably fatal and death usually occurs within three to five years of symptom onset, typically from respiratory failure. Therapeutic options remain limited despite significant research progress. Early diagnosis of ALS can be challenging due to the lack of pathognomonic symptoms and specific biomarkers [2]. Informative biomarkers to assist in earlier differential diagnosis, help determine prognosis, and serve as surrogates in therapeutic trials remain an urgent research priority in this field. 

Creatinine kinase (CK) is an enzyme involved in the transfer of high-energy phosphate in tissues with a large consumption of energy such as the skeletal muscle, heart and brain [3]. CK is widely used as a marker of muscle cell damage and is known to be moderately elevated in the sera of about 40% of ALS patients, either due to denervated muscle or metabolic changes in muscle fibers that occur as a consequence of the disease. Elevated CK levels also correlate with survival in ALS patients, although the existing literature presents conflicting results [4]. The utility of measuring CK values in ALS patients remains controversial. Of note, different CK isoenzymes consist of dimers of either type B or type M polypeptide chains. Although the MB isoenzyme (CK-MB) is expressed predominantly in the myocardium, skeletal muscles can contain up to 3% of CK-MB [5]. Historically, CK-MB serum levels have been used to evaluate patients with suspected acute myocardial infarction [6]. However, elevated serum levels of CK-MB can also be found in the serum as a result of muscle injury and other stressors such as surgical trauma [7]. Despite the wide availability, the use of serum CK-MB in the emergency evaluation of patients with chest pain is no longer recommended by evidence-based guidelines because CK-MB shows much lower specificity compared to high-sensitive cardiac troponins T (cTnT) or troponin I (cTnI) assays [8]. 

We have recently shown that cTnT, arguably reflecting neuromuscular involvement, but not cTnI, is commonly increased in the serum of patients with ALS, correlating with clinical severity as measured using the revised ALS Functional Rating Scale (ALSFRS-R) and thus could be of utility to distinguish the disease from ALS mimics or other neurodegenerative diseases [9]. While a few previous case reports have also reported elevations in CK-MB and CK-B subunit in the sera of patients with neuromuscular diseases including ALS [10,11], again presumably reflecting neuromuscular involvement, a role of CK-MB as a biomarker for ALS has never been formally explored.

In this study, we analyzed the serum CK-MB levels in a real-world cohort of ALS patients and their change over time to determine the utility of CK-MB as a possible biomarker of the disease. To explore the correlations between CK-MB and other factors, clinical and laboratory parameters were also reviewed. 

## 2. Results

**Figure 1:**
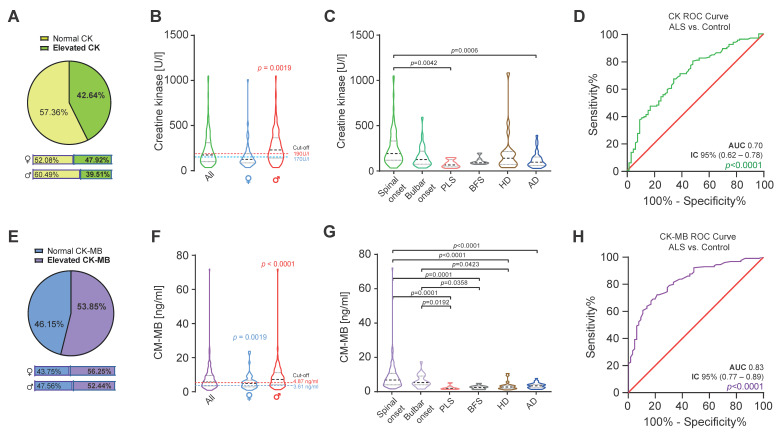
CK and CK-MB values in ALS patients. (**A**) Prevalence of elevated CK in ALS patients (n = 130). (**B**) CK levels are significantly increased in male patients in comparison to the cut-off value (Wilcoxon Test, W = 1328, and *p* = 0.0019). (**C**) CK values are significantly increased in patients with spinal-onset ALS in comparison to PLS and AD patients (Kruskal–Wallis test, H_5_ = 31.86, and *p* < 0.0016). (**D**) ROC curve illustrates CK’s (green line) association with ALS diagnosis in comparison to disease control (AUC 0.70, 95% CI [0.62 to 0.78], and *p <* 0.0001). (**E**) Prevalence of elevated CK-MB in ALS patients (n = 130). (**F**) CK-MB levels are significantly increased in female (Wilcoxon Test, W = 592.0, and *p* = 0.0019) and male (Wilcoxon Test, W = 2767, and *p <* 0.0001) patients in comparison to the cut-off values. (**G**) CK-MB values are significantly increased in patients with spinal and bulbar-onset ALS in comparison to PLS, BFS and HD patients (Kruskal–Wallis test, H_5_ = 69.64, and *p <* 0.0001). (**H**) ROC curve illustrates CK-MB (purple line) association with ALS in comparison to disease controls (AUC 0.83, 95% CI [0.77 to 0.89], and *p <* 0.0001). Red line the chance diagonal (AUC 0.5). Violin plots represent the actual distribution, median (dashed line) and quartiles (dotted line).

To evaluate CK and CK-MB levels in ALS and other neurological diseases, we reviewed data from 194 consecutive patients that frequented our department from January 2019 until August 2020. This group included 130 ALS patients and 64 disease control patients (primary lateral sclerosis [PLS, n = 8], benign fasciculations syndrome [BFS, n = 7], Huntington’s disease [HD, n = 20] and Alzheimer’s disease [AD, n = 29]). The baseline characteristics are provided in Table 1. The median age of ALS patients was 64.8 years (range 31.8–87.5) and the median disease duration was 1.75 years (range 0.3–11.2). ALS patients were predominantly male (75 male and 45 female) with clinical spinal onset. In this cohort, we found an elevation in CK levels above the upper reference limit in 42.64% of ALS patients (47.92% of females and 39.51% of males; Figure 1A). The median CK level in females was 137 U/L (range 36.0–1010.0 and cut-off value < 170 U/L) and in males was 234 U/L (range 35.0–1050.0 and cut-off value < 190 U/L). The median CK value was significantly higher than the reference value only in male patients (*p* = 0.0019; Figure 1B). CK-MB was elevated in the sera of more than half (53.85%) of ALS patients (56.25% elevation in females and 52.44% in males; Figure 1E). The median CK-MB levels in females was 4.85 ng/mL (range 0.8–23.7 and cut-off value < 3.61 ng/mL) and in males was 7.20 ng/mL (range 1.2–71.90 and cut-off value < 4.87 ng/mL; Figure 1F). The median CK-MB value was significantly higher in both sexes when compared to sex-specific cut-off values (female *p* = 0.0019 and male *p* < 0.0001; Figure 1F). In line with prior findings for CK [12], CK-MB was significantly higher in male than in female patients, especially in those with spinal-onset ALS (*p* = 0.0179).

**Figure 2:**
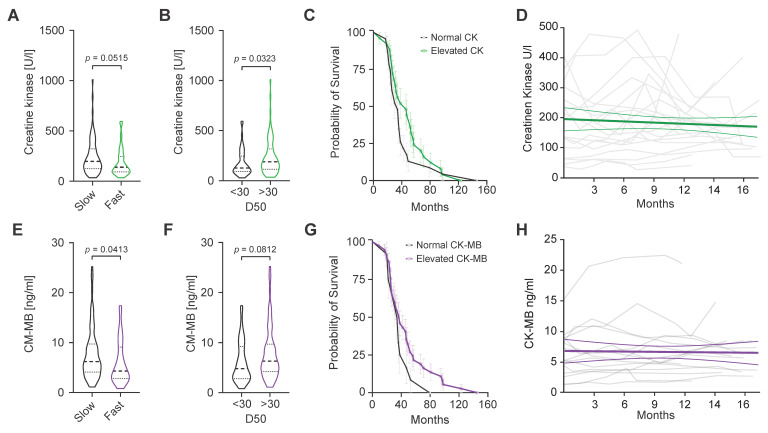
CK and CK-MB values, ALS aggressiveness, survival and progression. (**A**) Baseline CK values tend to be higher in slower ALS progressors (DR < 1/month). (**B**) ALS patients with lower overall disease aggressiveness (D_50_ > 30) presented higher CK levels (Mann–Whitney U = 1292 and *p* = 0.0452. (**C**) Kaplan–Meier survival curves from cases of ALS-related deaths in patients with normal and elevated baseline CK values. (**D**) Spaghetti plot of CK values (n = 20) versus time (months). Each grey line represents the longitudinal CK values of an individual patient. The green line represents a linear regression model. (**E**) Baseline CK-MB levels are significantly higher in slow ALS progressors (DR < 1/month, Mann–Whitney U = 1292, and *p* = 0.0413). (**F**) CK-MB levels tend to be elevated in ALS patients with lower overall disease aggressiveness (D_50_ > 30). (**G**) Kaplan–Meier survival curves from cases of ALS-related deaths in patients with normal and elevated baseline CK-MB values. (**H**) Spaghetti plot of CK-MB values (n = 15) versus time (months). Each grey line represents longitudinal CK-MB values of an individual patient. The purple line represents a linear regression model. Violin plots represent the actual distribution, median (dashed line) and quartiles (dotted line).

The routine data collected in our department included time and site of symptom-onset (spinal versus bulbar), and the degree of upper motor neuron involvement, allowing us to classify subgroups and make comparisons with other neurological disease control groups including PLS, BFS, HD and AD. The highest median CK level was found in the spinal-onset group (194.0 U/L and range 35.0–1050.0) but was only significantly different compared to the medians of PLS and AD groups (Figure 1C). Similarly, we found the highest median CK-MB level in the spinal-onset group (6.40 ng/mL and range 1.20–71.90), which was significantly different from the control disease groups (*p* < 0.0001; Figure 1G). This was also the case for the median CK-MB level in the bulbar-onset group (Figure 2G). To determine the diagnostic accuracy of the CK and CK-MB tests for discriminating ALS from other neurological diseases, we generated receiver operating characteristic (ROC) curves between ALS and our control disease group that included patients with PLS, BFS, HD and AD (n = 64). The CK ROC curve had an area-under-the-curve (AUC) of 0.70 (95% CI [0.62 to 0.78] and *p* < 0.0001; Figure 1D). The AUC for the CK-MB ROC curve was 0.83 (95% CI [0.77 to 0.89] and *p* < 0.0001; Figure 1H), remarkably similar to what we previously reported for cTnT [9]. 

CK elevations in ALS patients have been previously correlated with longer survival [13]. To assess a possible association between CK and CK-MB levels with ALS progression, we calculated the decline rate per month (DR) based on the initial ALSFSR score obtained in the first visit to our department and the time since disease onset (DR = [48-ALSFS-R score]/time since onset). Patients with a DR lower than 1 were considered slow progressors. Overall disease aggressiveness was indirectly estimated by calculating D_50_ as the time in months when 50% functionality was lost (ALSFRSR score of 24 from a maximum of 48) [14].

Patients with D_50_ lower than 30 were considered to have a higher overall disease aggressiveness. Additionally, we generated Kaplan–Meier survival curves from cases of ALS-related deaths (n = 49) after dividing the patients into groups based on normal or elevated CK or CK-MB (Figure 2C,G). CK levels tended to be lower in faster progressors when compared to slower progressors (*p* = 0.0515; Figure 2A). Similarly, patients with lower disease aggressiveness (D_50_ > 30) demonstrated higher median CK levels in serum compared to those with more aggressive disease (*p* = 0.0452; Figure 2B). In line with previous reports, patients with elevated CK levels showed a tendency towards prolonged survival (Figure 2C). Similarly, median CK-MB levels were significantly lower in faster progressors and tended to be higher in patients with slower progression (*p* = 0.0413; Figure 2E). Additionally, a tendency for longer survival in patients with elevated CK-MB was also observed (Figure 2G). In a subset of ALS patients (n = 20 CK and n = 15 CK-MB), longitudinal data allowed us to examine the dynamics of CK and CK-MB over time (Figure 2D,H). Spaghetti plots for longitudinal CK and CK-MB measures show variability between each repeated measurement over time without a clear increment or decrement. We calculated the change in CK and CK-MB over the longest available interval (minimum 30 days) as ΔCK/day (median −0.03000 and range −10.2–15.29) and ΔCK-MB (0.007300 and range −10.2–15.29), neither of which was significant. Additionally, a liner mixed model showed a negligible and non-significant average decrease in CK of −4.94^−7^ U/L and an increase in CK-MB levels of 7.41^−9^ ng/mL over 16 months (Figure 2D,H).

**Figure 3:**
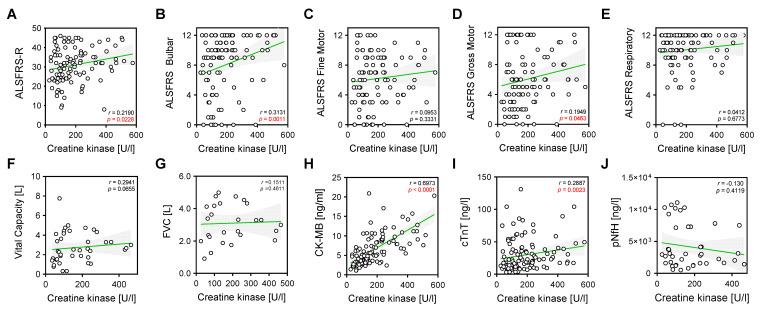
Correlations of CK with clinical and laboratory parameters. Correlation analyses were performed using nonparametric Spearman correlations (r). Curves were drawn using a linear regression model with an interaction term for CK in serum via (**A**) ALSFRS global score, (**B**) ALSFRS bulbar score, (**C**) ALSFRS fine motor score, (**D**) ALSFRS gross motor score, (**E**) ALSFRS respiratory score, (**F**) vital capacity (VC in liters), (**G**) forced vital capacity, (L) (**H**) CK-MB (ng/mL), (**I**) cTnT (ng/L), (**J**) pNfH (pg/mL) in CSF. Circles represent individual values; green lines represent the linear regression model and shaded areas represent 95% CIs.

**Figure 4:**
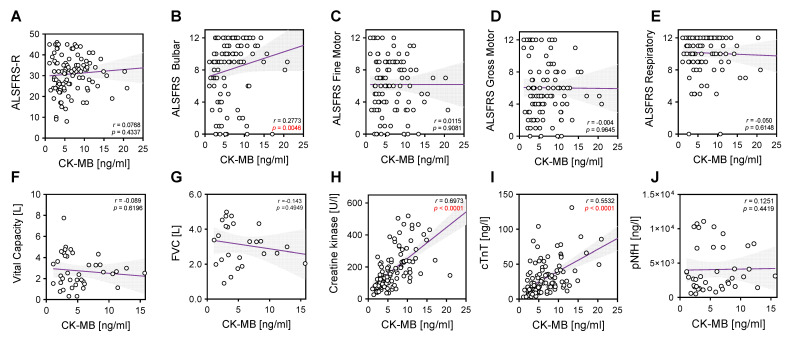
Correlations of CK-MB with clinical and laboratory parameters. Correlation analyses were performed using nonparametric Spearman correlations (r). Curves were drawn using a linear regression model with an interaction term for CK-MB in serum via (**A**) ALSFRS global score, (**B**) ALSFRS bulbar score, (**C**) ALSFRS fine motor score, (**D**) ALSFRS gross motor score, (**E**) ALSFRS respiratory score, (**F**) vital capacity (VC in liters), (**G**) forced VC (L), (**H**) CK (U/L), (**I**) cTnT (ng/L), (**J**) pNfH (pg/mL) in CSF. Circles represent individual values; purple lines represent the linear regression model and shaded areas represent 95% CIs.

To evaluate the association between CK and CK-MB levels, and clinical and laboratory parameters, we performed correlation analyses (Figure 3 and Figure 4). As expected, CK showed a strong positive and significant correlation with levels of CK-MB in serum (r = 0.6973 and *p* = 0.00001; Figure 3H) and positive mild but significant correlations with the total ALSFS-R (r = 0.219 and *p* < 0.0228; Figure 3A), ALSFS-R bulbar (r = 0.3131 and *p* = 0.0011; Figure 3B) and gross motor (r = 0.1949 and *p* = 0.0463; Figure 3D) sub-scores, as well as with cTnT levels in serum (r = 0.2887 and *p* = 0.0023; Figure 3I).

CK-MB values in serum correlated strongly with CK levels (r = 0.6973 and *p* < 0.0001; Figure 4H) and cTnT (r = 0.5532 and *p* < 0.0001; Figure 4I). Additionally, CK-MB demonstrated a significant positive correlation with the ALSFRS bulbar subcategory (r = 0.2773 and *p* = 0.0046; Figure 4B).

## 3. Discussion

There is an urgent need for informative biomarkers in ALS. In this study, we sought to investigate the role of CK and CK-MB elevations in the sera of ALS patients in potentially assisting earlier detection of the disease and determination of prognosis.

Our study showed that in contrast to total CK, but similar to what we previously reported for cTnT [9], CK-MB was elevated in more than half of all patients in this ALS cohort. CK-MB elevation in patients with spinal-onset ALS was significantly higher than in patients with other neurodegenerative diseases, but not significantly higher than in patients with bulbar-onset ALS. Similarly, a significant difference in CK-MB elevation was observed between ALS bulbar-onset and other neurodegenerative diseases. Elevated baseline CK values have been associated in some reports with longer survival in ALS [4,13]. In line with these results, we found a similar trend for baseline CK-MB serum levels (Figure 2G). Furthermore, patients with slower rates of decline in the ALSFS-R (<1 point/month) had a significantly higher baseline CK-MB, further supporting an association of CK-MB levels with longer survival (Figure 2E).

CK-MB serum level elevations also correlated with baseline clinical and laboratory parameters including cTnT (r = 0.55 and *p* < 0.0001), but lacks a correlation with pNfH in CSF, suggesting a role as a marker of muscle or lower motoneuron involvement. Of note, and in contrast to cTnT, CK-MB correlated with ALSFRS-R bulbar subcategory (r = 0.27 and *p* = 0.0046), potentially complementing the information gained with the use of cTnT in ALS diagnostic [9].

Longitudinal CK-MB values showed no significant temporal trend, suggesting that CK-MB does not change over the course of the disease and thus, unlike cTnT does not hold promise as a marker of disease progression, but holds value as a stable pathological marker [9,15]. The potential for utilizing CK-MB as a diagnostic marker in ALS was supported by the ROC curve analysis comparing ALS with control disease subjects, which demonstrated an AUC of 0.83 (*p* < 0.0001). This diagnostic performance improves to an AUC of 0.86 (*p* < 0.0001) when the cTnT values are added. This discriminatory power was even higher for patients with a spinal-onset of the disease or when compared to a control group of BFS and PLS patients (Table 2). It is then reasonable to speculate about an additive and complementary role of CK-MB to evaluate muscular and bulbar involvement in ALS when used together with other markers such as cTnT and neurofilaments (Nfs).

Although elevated serum CK levels are often reported in ALS and have been extensively studied in several ALS cohorts, the CK-MB isoform has surprisingly rarely been reported in patients with ALS. Since Smith et al. first reported serum CK-MB elevation in a single case of ALS in 1981, further reports have shown either normal or elevated CK-MB values in ALS patients [16,17,18]. However, a systematic study evaluating the role of CK-MB in diagnosis and manifestations of disease as well as the correlation with other putative ALS biomarkers was previously lacking. In the present study, we reported that serum CK-MB elevation occurred as frequently as an increase in serum cTnT in ALS patients, but lacks the temporal dynamic of the latter. CK-MB elevations were not associated with cardiac involvement. As has been suggested for serum cTnT, elevations of serum CK-MB may help bolster the diagnostic confidence when evaluating cases of suspected ALS [9].

The pathobiology underlying CK-MB elevations in ALS remains elusive and clearly needs further investigation. One could speculate that the gene coding for the CK subunit B is re-activated in the skeletal muscle during conditions of high chronic turnover of satellite cells, as has been shown for muscular dystrophies [19] or, alternatively, and less likely in our view, that motor neurons undergoing degeneration ectopically express the subunit M. Interestingly, higher baseline levels are found especially in patients with slower disease progression, suggesting that CK-MB elevation may reflect a protective response in ALS or could indicate a less aggressive more chronic disease process with longer periods of muscle denervation and isolated skeletal muscle fiber breakdown and necrosis [20]. Future investigation of muscle biopsies of ALS patients would help to test this hypothesis.

To the best of our knowledge, this is the first systematic study of CK-MB levels in a real-world cohort of ALS patients. This retrospective study demonstrated a complementary role and repurpose of a readily available routine laboratory parameter as a biomarker to evaluate neuromuscular and bulbar involvement in ALS. We note that the reported diagnostic performance of CK-MB may have been underestimated due to the lack of healthy controls, the relatively low number of cases investigated and the heterogeneity of the control group. Since we studied the levels of CK-MB in different stages of the disease progression, further controlled prospective studies should be performed to reaffirm the role of serum CK-MB as a diagnostic marker in ALS. Furthermore, the small subset of patients with longitudinal and mortality data are inconclusive in regard to the association with survival. On the other hand, serum CK-MB can be found elevated in other chronic muscle conditions such as inclusion body myositis [21] or polymyositis [22], both considered ALS mimics. CK-MB could underperform in the ALS diagnostic when measured in patients in which these chronic muscle conditions are suspected with concomitant chronic cardiovascular conditions.

The use of CK-MB to assess patients with suspected ALS could represent a great advantage as a rapid and inexpensive diagnostic aid, given its almost universal availability and years of experience in its application in laboratory medicine. Furthermore, the combination of this marker with a thorough clinical and electrophysiological examination or with other markers of neuronal (e.g., Nfs) and muscle damage (e.g., cTnT) could enable a safe diagnosis in early stages. In summary, this study shows that CK-MB could potentially aid in the diagnostic workup of ALS and help discriminate the disease from some ALS mimics and other neurodegenerative diseases. CK-MB levels also may provide useful prognostic information regarding disease aggressiveness as well as correlations with specific phenotypic presentations. An additional larger prospective study would be of value to support the potential for the use of serum CK-MB as a biomarker in ALS. 

## 4. Materials and Methods

### 4.1. Patient Cohort 

We retrospectively reviewed medical records of 130 ALS patients presented at the ALS Clinic of the Department for Neurodegenerative Diseases at the University Hospital of Bonn between January 2019 and August 2020. At the time of first contact, patients had either already been diagnosed with ALS or had progressive upper and lower motor neuron signs with clear evidence of neurogenic damage in needle electromyography examination [23], but the revised El Escorial criteria were not formally recorded [24]. For longitudinal data, the interval between each measurement was 90 ± 30 days, as patients were typically seen every 3 months. A total of 65 patients were included in this study as disease controls: 8 patients with primary lateral sclerosis (PLS), 8 with benign cramp fasciculation syndrome (BFS), 20 with Huntington’s disease (HD) and 29 with Alzheimer’s Disease (AD). All HD patients had one confirmed abnormally expanded CAG repeats in the huntingtin gene locus (>39 CAG repeats). AD patients fulfilled the CSF biomarker-based criteria according to the National Institute of Aging and Alzheimer’s Association (NIA-AA) Research Framework [25]. Disease progression in ALS patients was assessed using the Revised Amyotrophic Lateral Sclerosis Functional Rating Scale (ALSFRS-R), which includes bulbar functions, fine and gross motor skills, and respiratory function. The ALSFRS-R has a score of 0–40, with 0 representing the worst functional status and 40 representing the best.

### 4.2. Laboratory Markers

The assays of CK and its isoenzyme CK-MB were performed in the central laboratory of the Institute of Clinical Chemistry and Pharmacology at the University Hospital of Bonn. CK was determined with VIS-photometry using the cobas c702 module (Roche Diagnostics, Basel, Switzerland). Measurements were in units per liter “U/L” with cut-off values of 190 U/L for males and 170 U/L for females. CK-MB was measured via the electrochemiluminescence immunoassay method using the Elecsys^®^ CK-MB reagent and the cobas^®^ e801 module (Roche Diagnostics, Basel, Switzerland). Measurements were expressed in ng/mL with cut-off values of 4.87 [ng/mL] for males and 3.61 [ng/mL] for females. Troponin T was determined using the immunoluminometric assay. The measurement of pNfH from CSF was performed at the University Hospital of Ulm. Other laboratory parameters were also performed at the Institute for Clinical Chemistry and Pharmacology, University Hospital Bonn.

### 4.3. Statistical Analysis

The statistical analyses were performed using IBM’s SPSS 29.0.1.0 and GraphPad PRISM 9.5 software. Because CK and CK-MB presented a skewed distribution, data are presented as medians and nonparametric tests were used. For the two-way analysis of variance (two-way ANOVA), data were log-transformed to approximate normal distribution. Fisher’s exact test was used to assess the association between sex and clinical onset. Kruskal–Wallis test was used to analyze CK and CK-MB elevation between different diagnostic groups. Receiver operator characteristic (ROC) curve was used to evaluate the diagnostic properties. The Kaplan–Meier method was used to generate survival curves for patients with different serum levels of CK or CK-MB (normal or elevated) and compared using the log-rank test. The linear mixed effects model (LMEM) was used to assess the temporal trend of CK and CK-MB measured over time. Spearman’s correlation was used to explore the relationships between CK and CK-MB and other clinical characteristics and laboratory parameters. A *p*-value < 0.05 was considered significant.

## Figures and Tables

**Table 1:** Summary of patient characteristics.

	ALS						
	Spinal-Onset	Bulbar-Onset	PLS	BFS	HD	AD	*p*-Value
**Number**	102	28	8	7	20	29	
**Sex F:M ^a^**	33:69	15:13	8:0	1:6	11:9	18:11	0.0001
**Age, Years ^b^**	64.2 (31.8–87.5)	66.7 (45.5–81.0)	59.9 (46.9–72.5)	41.5 (27.8–53.1)	54.4 (24.1–69.1)	77.23 (34.6–87.0)	<0.0001
**Duration, years**	1.8 (0.3–11.2)	1.3 (0.3–5.1)	5.3 (0.5–25.2)	0.4 (0.0–10.7)	NA	NA	0.0119
**BMI (kg/m^2^) ^b^**	24.1 (16.5–39.9)	23.4 (16.1–27.4)	24.6 (18.7–36.9)	23.5 (20.9–35.9)	27.9 (20.2–35.4)	NA	0.2724
**ALSFRS-R ^b^**	34 (10–46)	32 (8–45)	34 (24–39)	48	NA	NA	<0.0001
**CK (U/L) ^b^**	194 (35.0–1050.0)	128 (35.0–593.0)	68.5 (29.0–150.0)	92.0 (74.0–199.0)	142.5 (39.0–1082)	99.0 (30.0–393.0)	<0.0001
**Females**	137 (36.0–1010.0)	93 (35.0–150.0)	68.5 (29.0–150.0)	84.0	117 (39.0–1082.0)	91.5 (38.0–228.0)	0.0832
**Males**	234 (35.0–1050.0)	154 (55.0–593.0)	NA	93.5 (74.0–199.0)	168 (64.0–231.0)	135 (30.0–393.0)	0.0030
**CK-MB (ng/mL) ^b^**	6.40 (1.20–71.90)	4.25 (0.80–17.1)	1.45 (0.90–4.80)	2.20 (0.80–4.40)	2.60 (1.00–10.20)	3.00 (0.90–7.30)	<0.0001
**Females**	5.10 (1.90–23.70)	3.80 (0.80–17.10)	1.45 (0.90–4.80)	2.80	2.40 (1.00–10.20)	2.60 (0.90–6.00)	0.0007
**Males**	7.60 (1.20–71.90)	5.00 (1.50–14.00)	NA	2.10 (0.80–4.40)	3.00 (1.40–5.20)	3.20 (0.90–7.30)	<0.0001
**Creatinine (mg/dL)**	0.70 (0.20–1.87)	0.73 (0.48–0.16)	0.73 (0.60–1.20)	0.83 (0.60–1.20)	0.92 (0.60–1.08)	NA	0.0298
**cTnT (ng/L)**	21.20 (3.0–300.0)	12.95 (3.0–77.90)	3.00 (3.00–9.30)	3.10 (3.00–6.90)	4.20 (3.00–13.90)	10.70 (3.00–30.0)	<0.0001
**pNfH in CSF (pg/mL) ^b^**	2219 (n = 50) (336–101725)	1959 (n = 12) (592.3–4205)	2541 (n = 2) (1400–3681)	90 (n = 7) (62.5–387)	246 (n = 3) (130.2–690.0)	NA	0.0002

^a^ χ^2^ test of independence. ^b^ Kruskal–Wallis test. AD, Alzheimer’s disease; ALSFRS-R, revised ALS Functional Rating Scale; BFS, benign fasciculation syndrome; BMI, body mass index; CK, Creatinine kinase; CK-MB, Creatine Kinase MB Isoenzyme; CSF, cerebrospinal fluid; F, females; HD, Huntington’s disease; M, males; NA, data not available; pNfH, phosphorylated neurofilament heavy chain. All data are presented in medians and range in parenthesis.

**Table 2:** Summary of ROC curves.

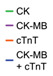	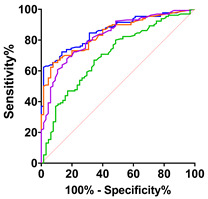				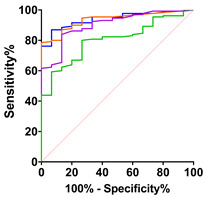				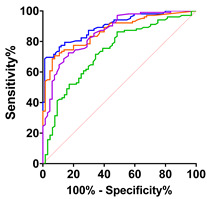				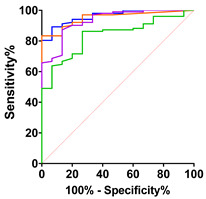			
	ALS vs. Control				ALS vs. PLC + BFS				Spinal-Onset vs. Control				Spinal-Onset vs. PLS + BFS			
	CK	CK-MB	cTnT	cTnT + CK-MB	CK	CK-MB	cTnT	cTnT + CK-MB	CK	CK-MB	cTnT	cTnT + CK-MB	CK	CK-MB	cTnT	cTnT + CK-MB
**AUC**	0.70	0.83	0.84	0.86	0.80	0.90	0.93	0.94	0.73	0.86	0.87	0.90	0.84	0.93	0.95	0.96
**SD**	0.04	0.03	0.03	0.026	0.05	0.03	0.02	0.02	0.04	0.03	0.03	0.03	0.04	0.03	0.02	0.02
**95% IC**	0.62–0.78	0.77–0.89	0.79–0.90	0.81–0.91	0.71–0.89	0.84–0.97	0.89–0.98	0.90–0.98	0.65–0.81	0.80–0.92	0.82–0.92	0.86–0.94	0.75–0.93	0.87–0.99	0.93–0.99	0.93–0.99
***p*-value**	<0.0001	<0.0001	<0.0001	<0.0001	0.0001	<0.0001	<0.0001	<0.0001	<0.0001	<0.0001	<0.0001	<0.0001	<0.0001	<0.0001	<0.0001	<0.0001

AUC, area under the curve; 95% IC, 95% confidence interval; SD, standard deviation.

## Data Availability

The raw data are unavailable due to restrictions of the German Federal data protection act.

## References

[1] Goutman S.A., Hardiman O., Al-Chalabi A., Chió A., Savelieff M.G., Kiernan M.C., Feldman E.L. (2022). Recent Advances in the Diagnosis and Prognosis of Amyotrophic Lateral Sclerosis. Lancet Neurol..

[2] Genge A., Chio A. (2023). The Future of ALS Diagnosis and Staging: Where Do We Go from Here?. Amyotroph. Lateral Scler. Front. Degener..

[3] Wyss M., Kaddurah-Daouk R. (2000). Creatine and Creatinine Metabolism. Physiol. Rev..

[4] Gao J., Dharmadasa T., Malaspina A., Shaw P.J., Talbot K., Turner M.R., Thompson A.G. (2022). Creatine Kinase and Prognosis in Amyotrophic Lateral Sclerosis: A Literature Review and Multi-Centre Cohort Analysis. J. Neurol..

[5] Dawson D.M., Eppenberger H.M., Kaplan N.O. (1965). Creatine Kinase: Evidence for a Dimeric Structure. Biochem. Biophys. Res. Commun..

[6] Saenger A.K., Jaffe A.S. (2008). Requiem for a Heavyweight: The Demise of Creatine Kinase-MB. Circulation.

[7] Prellwitz W., Neumeier D. (1979). Creatine-Kinase and CK-MB Isoenzyme Activity in Serum of Patients after Surgical Operations, Polytrauma and Other Damage to Skeletal Muscle. Clin. Biochem..

[8] Alvin M.D., Jaffe A.S., Ziegelstein R.C., Trost J.C. (2017). Eliminating Creatine Kinase–Myocardial Band Testing in Suspected Acute Coronary Syndrome: A Value-Based Quality Improvement. JAMA Intern. Med..

[9] Castro-Gomez S., Radermacher B., Tacik P., Mirandola S.R., Heneka M.T., Weydt P. (2021). Teaching an Old Dog New Tricks: Serum Troponin T as a Biomarker in Amyotrophic Lateral Sclerosis. Brain Commun..

[10] Jockers-Wretou E., Grabert K., Müller E., Pfleiderer G. (1976). Serum Creatine Kinase Isoenzyme Pattern in Nervous System Atrophies and Neuromuscular Disorders. Clin. Chim. Acta.

[11] Jockers-Wretou E., Vassilopoulos D. (1985). Serum Creatine Kinase B Subunit Levels in Neurogenic Atrophies. J. Neurol..

[12] Gibson S.B., Kasarskis E.J., Hu N., Pulst S.-M., Mendiondo M.S., Matthews D.E., Mitsumoto H., Tandan R., Simmons Z., Kryscio R.J. (2015). Relationship of Creatine Kinase to Body Composition, Disease State, and Longevity in ALS. Amyotroph. Lateral Scler. Front. Degener..

[13] Tai H., Cui L., Guan Y., Liu M., Li X., Shen D., Li D., Cui B., Fang J., Ding Q. (2017). Correlation of Creatine Kinase Levels with Clinical Features and Survival in Amyotrophic Lateral Sclerosis. Front. Neurol..

[14] Steinbach R., Batyrbekova M., Gaur N., Voss A., Stubendorff B., Mayer T.E., Gaser C., Witte O.W., Prell T., Grosskreutz J. (2020). Applying the D50 Disease Progression Model to Gray and White Matter Pathology in Amyotrophic Lateral Sclerosis. Neuroimage Clin..

[15] Kläppe U., Chamoun S., Shen Q., Finn A., Evertsson B., Zetterberg H., Blennow K., Press R., Samuelsson K., Månberg A. (2022). Cardiac Troponin T Is Elevated and Increases Longitudinally in ALS Patients. Amyotroph. Lateral Scler. Front. Degener..

[16] Rittoo D., Jones A., Lecky B., Neithercut D. (2014). Elevation of Cardiac Troponin T, but Not Cardiac Troponin I, in Patients with Neuromuscular Diseases: Implications for the Diagnosis of Myocardial Infarction. J. Am. Coll. Cardiol..

[17] Casmiro M., Graziani A. (2019). Serum Troponin T in Patients with Amyotrophic Lateral Sclerosis. Acta Neurol. Belg..

[18] Rosenbohm A., Schmid B., Buckert D., Rottbauer W., Kassubek J., Ludolph A.C., Bernhardt P. (2017). Cardiac Findings in Amyotrophic Lateral Sclerosis: A Magnetic Resonance Imaging Study. Front. Neurol..

[19] Tzvetanova E. (1978). Serum Creatine Kinase Isoenzymes in Progressive MuscularDystrophy. Enzyme.

[20] Carlson B.M. (2014). The Biology of Long-Term Denervated Skeletal Muscle. Eur. J. Transl. Myol..

[21] Lindberg C., Klintberg L., Oldfors A. (2006). Raised Troponin T in Inclusion Body Myositis Is Common and Serum Levels Are Persistent over Time. Neuromuscul. Disord..

[22] Tsung S.H., Huang T.Y., Lin J.I. (1982). Case Report CK-MB Isoenzyme in Patients with Polymyositis. Am. J. Med. Sci..

[23] de Carvalho M., Dengler R., Eisen A., England J.D., Kaji R., Kimura J., Mills K., Mitsumoto H., Nodera H., Shefner J. (2008). Electrodiagnostic Criteria for Diagnosis of ALS. Clin. Neurophysiol..

[24] Brooks B.R., Miller R.G., Swash M., Munsat T.L. (2000). World Federation of Neurology Research Group on Motor Neuron Diseases. El Escorial Revisited: Revised Criteria for the Diagnosis of Amyotrophic Lateral Sclerosis. Amyotroph. Lateral Scler. Other Mot. Neuron Disord..

[25] Jack C.R., Bennett D.A., Blennow K., Carrillo M.C., Dunn B., Haeberlein S.B., Holtzman D.M., Jagust W., Jessen F., Karlawish J. (2018). NIA-AA Research Framework: Toward a Biological Definition of Alzheimer’s Disease. Alzheimer’s Dement..

